# Perpetuation of gender discrimination in Pakistani society: results from a scoping review and qualitative study conducted in three provinces of Pakistan

**DOI:** 10.1186/s12905-022-02011-6

**Published:** 2022-12-22

**Authors:** Tazeen Saeed Ali, Shahnaz Shahid Ali, Sanober Nadeem, Zahid Memon, Sajid Soofi, Falak Madhani, Yasmin Karim, Shah Mohammad, Zulfiqar Ahmed Bhutta

**Affiliations:** 1 School of Nursing and Midwifery, Aga Khan University, Stadium Road, P.O. Box 3500, Karachi, Pakistan; 2 Department of Community Health Sciences, Aga Khan University, Stadium Road, P.O. Box 3500, Karachi, Pakistan; 3 Aga Khan Health Services, Karachi, Pakistan; 4 Center of Excellence Women & Child Health, Aga Khan University, Karachi, Pakistan; 5 Aga Khan Rural Service Pakistan, Gilgit, Pakistan; 6 Institute for Global Health, Karachi, Pakistan; 7 Department of Paediatrics & Child Health, Aga Khan University, Karachi, Pakistan

**Keywords:** Gender discrimination, Women discrimination, Gender, Pakistan

## Abstract

**Background:**

Gender discrimination is any unequal treatment of a person based on their sex. Women and girls are most likely to experience the negative impact of gender discrimination. The aim of this study is to assess the factors that influence gender discrimination in Pakistan, and its impact on women’s life.

**Methods:**

A mixed method approach was used in the study in which a systematic review was done in phase one to explore the themes on gender discrimination, and qualitative interviews were conducted in phase two to explore the perception of people regarding gender discrimination. The qualitative interviews (in-depth interviews and focus group discussions) were conducted from married men and women, adolescent boys and girls, Healthcare Professionals (HCPs), Lady Health Visitors (LHVs) and Community Midwives (CMWs). The qualitative interviews were analyzed both manually and electronically through QSR NVivo 10. The triangulation of data from the systematic review and qualitative interviews were done to explore the gender discrimination related issues in Pakistan.

**Results:**

The six major themes have emerged from the systematic review and qualitative interviews. It includes (1) Status of a woman in the society (2) Gender inequality in health (3) Gender inequality in education (4) Gender inequality in employment (5) Gender biased social norms and cultural practices and (6) Micro and macro level recommendations. In addition, a woman is often viewed as a sexual object and dependent being who lacks self identity unless being married. Furthermore, women are restricted to household and child rearing responsibilities and are often neglected and forced to suppress self-expression. Likewise, men are viewed as dominant figures in lives of women who usually makes all family decisions. They are considered as financial providers and source of protection. Moreover, women face gender discrimination in many aspects of life including education and access to health care.

**Conclusion:**

Gender discrimination is deeply rooted in the Pakistani society. To prevent gender discrimination, the entire society, especially women should be educated and gendered sensitized to improve the status of women in Pakistan.

## Background

Gender discrimination refers to any situation where a person is treated differently because they are male or female, rather than based on their competency or proficiency [1, 2]. Gender discrimination harms all of society and negatively impacts the economy, education, health and life expectancy [1, 2]. Women and girls are most likely to experience the negative impacts of gender discrimination. It include inadequate educational opportunities, low status in society and lack of freedom to take decisions for self and family [1, 3].

Likewise, gender discrimination is one of the human rights issues in Pakistan and is affecting huge proportion of women in the country [1, 2]. In Pakistan, nearly 50% of the women lacks basic education [4]. In addition, women in Pakistan have lower health and nutritional status. Furthermore, most of the women are restricted in their homes with minimal or no rights to make choices, judgments, and decisions, that directly affect their living conditions and other familial aspects [2]. In contrast, men are considered dominant in the Pakistani society [5]. This subordination of women has negative influences on different stages of women’s life.

## Methods

### Study design

The mixed method study design was used. Systematic review was done in phase one and qualitative interviews; in-depth interviews (IDIs) and focus group discussions (FGDs) were conducted in phase two.

### The objective of the systematic review

To map a broad topic, gender discrimination/inequality research in Pakistan including women undergoing any form of intimate partner violence.

### Systematic review

**Fig. 1:**
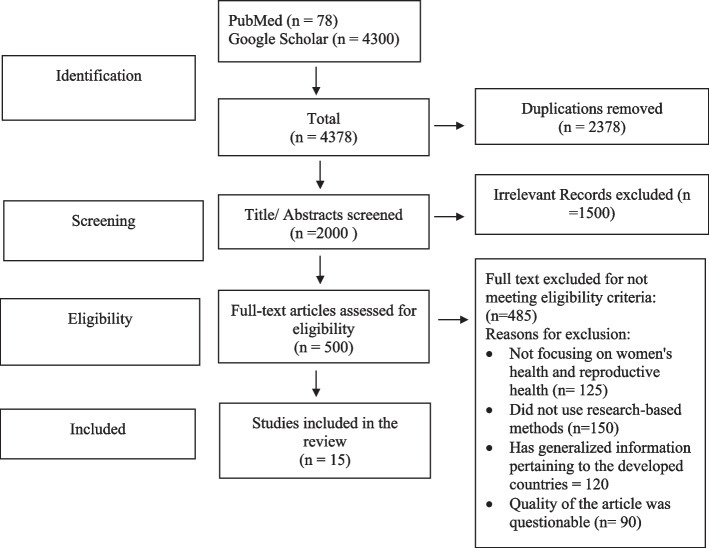
PRISMA Diagram to select the final articles

The three authors (TSA, SSA and SN) independently performed an extensive literature search using two databases: PubMed and Google Scholar and reports from organizations such as WHO and the Aurat Foundation. Quantitative and Boolean operators were used to narrow down the search results. The following keywords and phrases were used: Intimate partner violence (IPV), domestic violence, violence against women, domestic abuse, spousal violence, and Pakistan. Articles from 2008 to 2021 were assessed. The selection criteria of the articles included: women undergoing any form of IPV (physical, psychological, and sexual); quantitative study design; English as the publication language; and articles in which Pakistan was the study setting. The shortlisted articles were cross-checked by two of the authors (TSA, and SN) for final selection. The quality of the selected articles was reviewed using a STROBE (Strengthening the Reporting of Observational Studies in Epidemiology) checklist, which ensured all articles followed a structured approach, including an introduction, methodology, results, and a discussion section. It was also determined that all selected articles are published in peer-reviewed journals and have been used nationally or internationally. The Preferred Reporting Items for Systematic Reviews and Meta-Analyses (PRISMA) chart was used for study selection (Fig. 1).

The selected articles were approved by one of the authors (TSA), who is an expert in the field of IPV. Articles were excluded: (i) If the study was not conducted in Pakistan; (ii) Studied spousal violence against men and (iii) Domestic violence involving in-laws or other family members. Furthermore, from the selected articles, the data were extracted by 3 authors (TSA, SSA, SN) by carefully studying the methodology and results. The methodology was entered into an extraction template in which location was summarized including the study design and sample size in the articles. The results covered: (i) The title, (ii) Authors, (iii) Publication year, (iv) Objectives of the research, (v) Population and Setting, (vi) Research design, (vii) Data collection methods, (ix) Results, (x) Perpetuating factors (xi) Recommendations and (xii) prevalence of Intimate Partners Violence (IPV) faced by women, which was further categorized into: (a) Psychological/emotional violence, (b) Physical violence, (c) Sexual violence, (d) Both combined and (e) Violence of any other type.

### Qualitative data collection

#### Participants selection

Purposeful sampling was done to recruit the participants for qualitative data collection. Participants included groups of married men and women aged between 18 to 49 years, groups of unmarried adolescent boys and girls aged between 14 to 21 years, and groups of healthcare professionals (HCPs), comprising of doctors, nurses, Lady Health Visitors (LHVs), Lady Health Workers (LHWs) and Community Midwives (CMWs). Ethics approval was obtained from the Aga Khan University, Ethics Review Committee.

#### Study sites

The selected study sites included two districts from Chitral (Upper and Lower Chitral), six districts from Gilgit (Gilgit, Ghizer, Hunza, Nagar, Astore, and Skardu), and two districts from Sindh (Matiari and Qambar Shadadkot). The following are the details of the data collection (Refer Table 1).

**Table 1:** Details of Qualitative assessments of community members and Health care professionals

Districts	Qualitative assessment type	Study participants
**Gilgit Baltistan** (Skardu, Astore, Ghizer, Gilgit, Hunza, Nagar)	IDIs (*n* = 18)	Gynaecologist (*n* = 4), Hospital administrator (*n* = 1), CMW (*n* = 4), HOD (*n* = 4) and LHV (*n* = 5).
	FGDs (*n* = 32)	Adolescent girls (*n* = 6), Adolescent Boys (*n* = 8), Married Men (*n* = 6), Married women (*n* = 6) and HCP (*n* = 6)
**Chitral** (Upper and Lower Chitral)	IDIs (*n* = 8)	Gynaecologist (*n* = 2), Hospital administrator (*n* = 2), CMW (*n* = 1), LHV (*n* = 2) and Head nurse (*n* = 1).
	FGDs (*n* = 14)	Adolescent girls (*n* = 3), Adolescent Boys (*n* = 4), Married Men (*n* = 2), Married women (*n* = 3) and HCP (*n* = 2)
**Sindh** (Matiari, and Qambar Shadadkot)	IDIs (*n* = 11)	Gynaecologist (*n* = 3), Hospital administrator (*n* = 1), CMW (*n* = 1), LHV (*n* = 2), Staff nurse (*n* = 1), HOD (*n* = 1), LHW (*n* = 1) and midwife (*n* = 1).
	FGDs (*n* = 11)	Adolescent girls (*n* = 2), Adolescent Boys (*n* = 2), Married Men (*n* = 2), Married women (*n* = 3) and HCP (*n* = 2)

#### Data collection

Data were collected by conducting (IDIs) and (FGDs). The IDI and FGD interview guides were developed specifically for the study and reviewed based on the literature. IDIs were conducted with the healthcare industry administrators, Heads of the Departments (HODs), and HCPs of private and government health settings, including gynaecologists, LHWs, LHVs, and CMWs. The IDI interview guides comprised of the questions related to knowledge, sources of information, and attitudes regarding gender-based discrimination (how each gender is perceived in society and how physical and social differences in the roles of males and females affect an individual or society). The IDIs were conducted in Urdu and local language. The interviews were audio-recorded. Each IDIs lasted for 45–60 minutes.

Likewise, the FGDs were conducted using different interview guides, which were designed to assess the perception of adolescent girls and boys, married men and women and health care workers regarding gender discrimination in the society (perceptions of masculinity and femininity, and gender role expectations of a society). The FGDs were conducted in Urdu and local language. The interviews were audio-recorded. Each FGDs lasted for 60–120 minutes.

#### Data analysis

All interviews were audio recorded and transcribed in English. Training was provided to the data collectors, and they were supervised by the authors throughout the process to ensure transcriptions are written accurately and correctly, representing the actual data collected during interviews. Thematic analysis was carried out in four different steps. Firstly, manual analysis was done by the research team where transcriptions were thoroughly read, and codes were identified. These codes were combined according to their contextual similarity which followed the derivation of categories, based on which, themes were developed. Secondly, similar manual analysis was conducted by an expert data analyst. Thirdly, analysis was conducted using QSR NVivo 10. In the final step, all three analyses were combined and verified by the research team followed by the compilation of results.

#### Data integrity

To maintain the credibility or truthfulness of the data, the following strategies were used: (1) Prolonged engagement: Various distinct questions were asked related to the topic and participants were encouraged to share their statements with examples, (2) Triangulation: Data was analyzed by the author, expert data analyst and through QSR NVivo10, (3) Persistent observation: The authors read and reread the data, analyzed them recoded and relabeled codes and categories and revised the concepts accordingly, and (4) Transferability: The ability to generalize or transfer the findings to other context or settings, was ensured by explaining in detail the research context and its conclusions [6].

#### Ethical considerations

Ethical approval was obtained from the ethics review committee (ERC), Aga Khan University. The ERC number is 2020-3606-11,489. To ensure voluntary participation of the study participants both verbal and written consent were obtained. For those who were younger than 18 years of age were given written assent, and their parent, or guardian’ verbally consented due to literacy issues. In addition to anonymity of the study participants were maintained by assigning codes to the study participants. To avoid loss of data, interview recordings were saved on a hard drive and in the email account of the author. The data on hard copies such as note pads used during IDIs and informed consents were kept in lock and key. All the data present in hard copy was scanned and saved in the hard drive with password protection. To ensure confidentiality, only the authors had access to hard and soft data of the study.

## Results

### Systematic review

The studies selected were scrutinized to form a data extraction template with all the relevant data such as author, publication year, study title, purpose, design, setting, sampling, main results, perpetuating factors, and recommendations (Refer Table 2, provided in the attachment). Most of the 20 studies included in the review were conducted in Pakistan however the most frequent study design was cross-sectional (*n* = 9) followed by narrative research based on desk reviews (*n* = 8), one was a case study, and two were cross-country comparison by using secondary data. Four studies were conducted in Province Punjab, three studies were conducted in KPK, and one in both KPK and Punjab. Only one study was conducted in Sindh province. The remaining used whole Pakistan in systematic review. The maximum sample size in a cross-sectional study was (*n* = 506). Six major themes have emerged from the review which included (1) Status of Women in Society (2) Gender Inequality in Health (3) Gender Inequality in Education (4) Gender Inequality in Employment (5) Gender Biased Social Norms and Cultural Practices (6) Micro and Macro Level Recommendations.

**Table 2:** Systematic review

S #	First Author, Year & Title	Objectives	Population & Setting	Research Design & Data Collection	Main Results	Perpetuating factors	Recommendations
1	Iqbal (2012) Gender Discrimination: Implications for Pakistan Security [1]	• To describe the gender discrimination in Pakistan • To describe gender discrimination in education and employment • To discuss gender and human security	Pakistan	Desk review, narrative research	Gender balance can be improved by enhancing non-traditional security Gender discrimination against females can be reduce by providing them equal chances at the workplace and in society.	**Social Cultural** -Patriarchal society -Honor killing -low social, economic, and political status in society -DV is personal matter **Education:** -Cultural boundaries -Co-education -poor physical environment and lack of fundamental services in education sector -limited opportunities for rural girl for education **Employment:** **-**low literacy rate -Marriage -Childbearing -unfavorable work environment -discrimination in male and female pay scale -Low promotion -Fewer female in decision making and leading position.	Eliminate discriminatory attitude of the society towards the women. The constitution of Pakistan guarantees equal rights to all citizens and make sure the forceful implementation. Govt must start mass media awareness campaign on gender discrimination in employment. Female employees must be informed about their rights and privileges. NGOs should come forward to support the victimized female employees to get justice. -Govt should compilation annual reports of Human resource in Govt. and private sector. More investment on female education
2	Delavande (2013) Gender Discrimination and Social Identity: Experimental Evidence from Urban Pakistan [3]	To find out the discrimination against females regarding gender and social class	*N* = 2836 male and female students from Islamabad, Rawalpindi and Lahore	Experimental design -Survey questionnaire	Nature and intensity of gender discrimination is not same in all educated class Higher socioeconomic status females are not discriminated and are even favored in some instances by males Women of lower socioeconomic status are discriminated against by certain groups of men	-Low socioeconomic status -Discrimination because of Social Identities (Class, ethnicity, race)	-Push for policies favoring economic development, such as education policy, will lead to an improvement in women’s rights and status. -Male education can play an important role in discrimination as educated males tend to discriminate less -Gender equitable child rearing practices at home including education to boys.
3	Alam (2011) Impact of Gender Discrimination On Gender Development and Poverty Alleviation [7]	To know the main reasons and areas of gender discrimination and its impact on development and poverty alleviation	*N* = 50 25 Male and 25 female selected from purposive sampling from Peshawar	Cross-sectional study Survey questionnaire and Interview	Gender disparity exists in targeted geographies. women have no or low share in income / earnings of the family Women are more vulnerable to poverty; women share more burden of productive and household work. Women are not given equal opportunities in jobs Gender inequalities hinder poverty alleviation. Lack of women participation in development and women have no political and propriety rights. Lack of skill enhancement opportunities for women would affect poverty alleviation and gender development	No equal status of women in family as she didn’t contribute to family earning. Female were not allowed to work outside home. Decision makers are male in household. No equal opportunity in higher education for females Old traditional role of male and female hindering the women development Sex segregated occupational choices; females are only in teaching. Females are economically dependent on man Exclusion from development and it affects poverty alleviation.	Equal educational opportunities and quality education should be provided to women for different jobs. Women should be given equal rights and power of decision-making. Equal opportunities of Participation to bring equality in resources distribution Household and productive burden should be share equally that may help in poverty alleviation. Skills training facilities should be available for females Equal opportunity in family affairs. Need of policy formation focused on women empowerment for poverty alleviation and gender development.
4	Bukhari (2019) Gender Inequality: Problems & Its Solutions In Pakistan [4]	To highlights the practices of gender discrimination, identifies the problems and its solutions in Pakistan	Pakistan	Desk Review, narrative research	Female discrimination is present due to socio-cultural norms in Pakistan. Gender discrimination mostly influence by tribal, feudal and religious social formations. Financial problems are a major concern for educating the women Girls face harassment and criticism from society while traveling - Rural females are suffering more in terms of getting education. -Government seats mostly occupied by males.	Male dominancy Lack of participation in discission making Social cultural norms and inhuman cultural practices Co-education and boarding facilities are hindrances in girl education.	Provide equal status and opportunities to women in society Empower women by promoting education, training, and professional development Ensure the Health, safety, and well-being of all women and men. Protection against domestic violence and legislation against Domestic violence Equal representation in public and private sector
5	Ejaz (2011) Gender Discrimination and The Role of Women in Pakistan [2]	To review the practices of gender discrimination, its contributing factors and recommendation to make women powerful in society.	Pakistan	Desk review, narrative research	In Pakistan as Muslim society gender discrimination, it is in the most hideous form Islamic values and laws were fabricated and presented as subliminal messages. Some writers use their venomous words to disgrace and degrade women openly Girls passively wait to accept their destiny with anticipation of discrimination, injustice, and unequal distribution of human rights.	Patriarchal society Low education level. Discriminatory laws in Pakistan Unequal Sexual division of labor Reproductive activities Cultural Norms and practices Early and forced marriages Fabricated Islamic messages Brainwashing at toddler age and intergenerational passing of submissiveness by mothers Stereotype role of women in media.	Women themselves must understand their proper status in the society Awareness of rights are important for both male and female and they need to respect each other, Efforts can be made to make people understand the translation of the holy Qur’an. Unbiased teaching of Islam to provide women with appropriate knowledge making them all-powerful in the society
6	Ferrant (2016) Does gender discrimination in social institutions matter for long-term growth? [5]	To estimate the potential income gains associated with gender parity and the cost of the current level of discrimination.	Multicultural study including Pakistan	Cross country secondary data analysis	Gender-based discrimination in social institutions obstructs the economic development and income of the country The association between low economic development gender disparity is stronger in low-income countries. Per-Capita income of both and females are reducing because of gender discrimination High income countries have lower levels of gender-based discrimination in social institutions	Less access to education	Gender disparity in social institutions need to be integrated in growth analysis of the country. More investment on girl education and trainings. Social institutions should be gender responsive and have gender-transformative policies. Identify the bottlenecks of gender parity and unlock the growth potential of social institutions. Dire need to identify which type of institution have greater effect and which intervention will be needed to reduce the gap.
7	Mahmood (2012) Gender-Specific Barriers to Female Entrepreneurs in Pakistan: A Study in Urban Areas of Pakistan [8]	To determine gender-specific barriers and its impact for female entrepreneurs in Pakistan	*N* = 160 Females from Faisalabad Multan Sukkur Sargodha Sialkot	Cross sectional Study Self-administered survey questionnaire and in-depth interviews	Female entrepreneurs are very important to economic and social development in Pakistan. Female entrepreneurship is a grater tool to transform economy of Pakistan. Female participation in entrepreneurial activities is not only empower them economically but also have positive social influences on women themselves, their families, and their social environment.	**Capital unavailability** Lack of finance Poor knowledge about loan process discrepancy in property rights **Culture and local customs** Restriction on mobility Communication gap with men Limited decision making role models and guiders Lack of education **Gender discrimination** Men’s hold on market Childcare burden Family pressure Discrimination Harassment	For the development of full potential of women there is a need to understand recognize and support them. Make sure the women participation in economic activities to achieve sustainable goals. Ensure the provision of finance.
8	Tarar (2014) Patriarchy, Gender Violence and Poverty amongst Pakistani Women: A Social Work Inquiry [9]	To find out discrimination and violence against women in Pakistan and its interplay with patriarchy and poverty. To examine how the patriarchy, us a tool to	*N* = 52 Females living in Shelter home Punjab Pakistan	Descriptive qualitative, Primary research paper Interviews	Women are vulnerable to different types of violence, and this vary by class There is a relationship between patriarchy, poverty, and gender violence Poverty appears to be the direct cause of gender violence	Poverty Patriarchal society Economical dependency less access to education Husband’s drug addiction	Understanding of psychological, social, and gender aspects of violence to eradicate poverty. Educate and empower women to promote economic, social, and gender equalities as well as to improve the social structure. Empowerment oriented social workers to focus on victim’s strengths, adaptive skills, and competencies to resolve their problems.
9	Kazimi (2019) Mothers Role and Perception in Developing Gender Discrimination [6]	To analyze role of mothers in developing child personality and social behavior. To explore mother role in developing gender discrimination.	*N* = 183 Mothers Residing in urban and rural Sindh province selected through purposive and random sampling	Mix Method research Design Survey Questionnaire and narrative inquiry	Urban and rural mothers both have discriminatory behavior Mother’s attitudes towards boys and girls are highly biased and both treated differently. Mother’s discriminative behavior helping in promoting and developing gender discriminated behavior in society especially in rural areas -Mothers are not aware that they are harvesting the gender gap in society with their discriminative behavior -Mother can play a role to reduce gender discrimination.	Discriminatory child rearing practices Son preference Pro male behavior Patriarchy Lack of participation in decision making. Violation of property rights.	Women education and adult education programs for the awareness of the consequences of mother behavior Provision of equal rights and opportunities for both men and women Mother education for gender parity in practices. Schools can play a role and should focus on this problem and guide parents. Use of Media especially television for developing awareness among mothers
10	Atif (2016) Son Preference in Pakistan: A Myth or Reality [10]	To study the relationship between various demographic characteristics and desire for son	*N* = 506 Married females of Childbearing age from Peshawar, Pakistan	Cross-sectional Study Survey questionnaire	Significant relation between sons desired and female education, husband’s education, and socioeconomic class. No significant impact of religion on desire for sons. Significantly higher desire for infinite number of sons than daughters, or want at least one son	Dowery Lack of social support	Couple counseling to reduce gender bias Girls should be given equal chances to be wishfully borne by their parents, and live with equal right
11	Rabia (2019) Gender Inequality: A Case Study in Pakistan [11]	To explore the perception of male and females on gender inequality in Pakistan	*N* = 357 Male and female College students from Sialkot Punjab, Pakistan	Cross sectional Study Survey questionnaire	There is gender discrimination in home, in education and at workplace. Parents give more importance to boys as compared to girls Women can work outside their home if the income is not adequate. Mostly backward area people have controversial thoughts about women and more women experienced gender inequality -Honor killing and practices like acid attacks are very common.	-Son preferences -Child rearing practices -Social norms	Government and public both should work to promote education and professional development for women. Removal of discriminatory attitude of the society towards the women. Encourage women participation in economic growth. Equal opportunity to both men and women at home, education and workplace. Ensure health, protection, and happiness for all women Provide protection against domestic violence and mistreatments
12	Shah (2012) Girl Education in Rural Pakistan [12]	To identify the barriers of girl’s education in rural areas and recommendations to eliminate these barriers.	*N* = 35 Stakeholders in education including parents and teacher from a rural Punjab, Pakistan	Case study In-depth interviews Field notes/ Observations	Muslim girl should be brought up as she can become good wife, daughter, sister, mother and wife. Poverty and lack of public education is considerably lower in rural areas	Cultural norms Child rearing practices Patriarchy Religious leaders Poverty Co-education Distance of school Child labor Early Marriages Domestic role of female Lower status of female in society Absence of female teachers	Creates proactive policies, essential infrastructures, and necessary resources Ensure the safety and protection of girls. Awareness and community sensitization for girl education. Create girl’s enrollment campaigns at local level with some incentives on enrollments Partnership between all stakeholders at all levels.
13	Hamid (2011) An Analysis of Multi-dimensional Gender Inequality in Pakistan [13]	To investigate multi-dimensional gender inequalities faced by women in the socio-economic and political scenario of Pakistan	Pakistan Comparison made between male and female	Desk review	Women in Pakistan are suffering from multi-dimensional inequality The dimensions of family, women identity, health, education, women access to economic resources, employment and participation in politics contribute significantly to the discrimination of women Discrimination against women starts from the very beginning Poor and inadequate health and nutrition facilities for antenatal women and SRHR Very low women employment in non-agricultural sector and mostly are in informal sector where wages are very low	Patriarchy Child rearing practices Poor and inadequate services Male control over financial and physical assets Son preferences Lack of participation in decision making **Low education:** Poverty domestic and farming Responsibilities low access to schools Early marriages Socio-cultural practices. Unsafe places **Low employment:** Lower education Social cultural norms constraints on women’s mobility	Provision of opportunities to women in the form of education, better health, possession of assets, employment and for participation in politics -Legislation of laws to protect women rights Creation of gender friendly enabling environment for economic, social, and political participation. Parents sensitization on right of female child and make sure the active participation of girls at all levels. Provision of Child Friendly Schools, trainings and skills program for females Introduction of uniform wages and salaries for both gender
14	Bhattacharya (2014) Status of Women in Pakistan [14]	To evaluate the role of women in relation to various layer of state and society.	Pakistan	Desk Review Narrative	Quranic principles are frequently avoided and twisted, especially regarding the status of women Guardians of the faith take them as customary to ‘punish’ women and ostracize them Islam not only gives basic equality to women but also equal legal rights Patriarchy has caused a total disregard for women in Pakistani society. Feudal system in society has also caused severe antipathy towards women.	Medieval cultural practice Women ignorance about their rights. Anti-Islamic cultural practices and traditions (Haq Bakhshish) Tribal judicial system Institutional and individual violence Patriarchy Female viewed as inferior. Feudal system Violence at home Honor killing Selling of Bride Dowery Acid Attack	Frequent training and awareness programmers are needed to be arranged periodically to make women aware of their rights Public, as well as private sectors, should be established to provide job opportunities to rural women so that they can earn income Laws need to be reviewed and adequate implementation of laws to provide a safe and secure environment for women Education and awareness of men and women both are very important
15	But (2016) Social Policy and Women Status in Pakistan: A Situation Analysis [15]	To review status of women and social policies in Pakistan	Pakistan	Desk Review Descriptive research	Govt. is unwilling to improve women status in country. Women face poverty, lack of access to health and education services There is unavailability of domestic policies to protect women from violence and gaps in implementation The is a high ratio of gender inequality and sone preference in rural and urban areas - Property rights is often transferred to the male members of the society -Political instability worsen the situation and increase the gender gap.	lack of gender friendly policies and implementation. Patriarchy No property rights Gender Based Violence- No equal rights Sexual abuse/rape **Health** inaccessibility of health facilities Cultural norms and taboos poverty-lack of services domestic violence **Education** -Child Marriages -lack employment opportunities -sex segregation -school distance Domestic chores Co-education Son preference Parents illiteracy **Employment** -Narrow job opportunities -home responsibility -small industries -traditional division of labor -temporary positions	Provision of equal rights for women. Awareness and education to both males and females about equal rights Actual Islamic practice should be followed. Proper allocation of budget for women empowerment
16	Khurshid (2020) Analyzing the Impact of Gender Inequality on Economic Development in Pakistan: ARDL Bound Test Cointegration Analysis [16]	To assess the relationship between gender inequality and economic development and also analyze the short term and long-term impact of gender inequality on economic development	Pakistan	Time series Analysis	Gender inequality delayed both long term and short-term economic growth. Gender inequality has an inverse relationship with economic development. State of gender equality is abysmal in Pakistan that decreased their decreased roles in different spheres of life.	Inflation rate has negative impact on economic growth. Low women Literacy is the primary indicator of economic delayed Interest rate negatively but highly significant impact on economic development.	Female access to higher education and health can increase economic growth. Reduce in gender discrimination needed in education sector. Trade has a positive effect on economic growth. Holistic approach can overcome the consequences of gender inequality and crucial for economic development. Government should make policies to increase job opportunities for female.
17	Mahmood (2021) Sociological Analysis of Women’s Empowerment in Pakistan [17]	To explore the relationship between type of employment, employment status, occupation, and empowerment in Pakistan. To explore the relationship between demographics variables, financial contribution and empowerment in Pakistan.	Pakistan	Descriptive research	Women access to services and her contribution to extent family income has significant role to eliminate gender discrimination Mostly females are motivated to opt limited occupations. Women are less empowered in all fields of activities	Lack of access to services Lack of participation in economic activities and have limited job opportunities. Lack of empowerment in all fields. Lack of authority, Mobility restrictions and less resource control Domestic violence Patriarchy	Create more professional markets to empower women particularly for rural areas women. Include women in earnings Access to education Remove all the barriers that hinder women empowerment Need structural changings for gender equality Develop professional competencies by continue trainings and support. Educate women to know their rights Practice right to take decision
18	Mumtaz (2019) Frequency and Psychosocial Determinants of Gender Discrimination Regarding Food Distribution among Families [18]	To study the psychosocial factors causing gender discrimination regarding food distribution among families	Pakistan *N* = 50 females	Cross sectional study design	Higher discrimination in food distribution due to large family size Food discrimination 4 times higher in females Low income contributes to higher discrimination Maternal education lead reduction in food discrimination	Large family size Low income Male earners Lack of control on capital	Policies to promote Female education Create earning opportunities Provision of foods for poor families Awareness raising on print and social media for gender parity
19	Ahmed (2019) Impact of Gender-Specific Causes on Women Entrepreneurship: An Opportunity Structure for Entrepreneurial Women in Rural Areas [19]	to assess impact of gender-specific causes and factors on women entrepreneurship in rural areas of Pakistan.	*N* = 342 women and men from rural areas from *Sindh* *Panjab* *KPK* *Balochistan,* Pakistan	Cross sectional study	Gender discrimination is one of the major causes affecting women’s entrance in the process of entrepreneurship Men seen as bread earner primary source of income and woman seen a key caretaker, inside the home Safety, security issues and lack of education have further contributed to gender discrimination	Gender discrimination practices Lack of education Lack of access to capital and family support Inferior female status, lack of women’s role in decision making and early marriages Monopoly of men in the market Negligence of government	Create supportive environment for female entrepreneurs Provide entrepreneurial opportunities Provide public safety nets to improve women status as entrepreneur Offer vocational trainings Cash transfer for girls schooling Flexibility in working hours for females
20	Ahmad (2020) Men’s Perception of Women Regarding the Internet Usage in the Khyber Agency Pakistan: An Exploratory Study [20]	To investigate the gender discrimination in the use of the internet in KPK To explore the reason of gender discrimination	*N* = 100 15–29 years old youth KPK, Pakistan	Mix method approach	Digital divide in internet usage promotes gender discrimination KPK women suffered from gender discrimination Women has lack of access to the internet and the usage of the internet compared to men Most of the people are far from digital devices Men has decision making power for health education, marriage etc. Internet use not allowed to female by male members of the family because of misuse and content on internet. Internet seems threat to traditional Parda system (Veil)	Patriarchy Deprivation from digital world Stereotypical discourse of women Traditional values Religious restrictions Lack of participation in decision making	Awareness raising Development of local women organizations Promote women education Campaign on the benefit of internet by Govt. and NGOs Dialogue with male members of the society IT related scholarships for females Women empowerment campaigns Women access to technology

#### Status of a woman in the society

The Pakistani women often face gender inequality [13]. Women are seen as a sexual object who are not allowed to take decision for self or their family. However, the male is seen as a symbol of power. Due to male ownership and the patriarchal structure of the Pakistani society women are submissive to men, their rights are ignored, and their identity is lost. Out of twenty, nine studies reported that a female can not take an independent decision, someone else decides on her behalf, mainly father before marriage then-husband and son [1, 3, 4, 6–8, 13]. The three studies report that women are not allowed to participate in elections or have very limited participation in politics. Furthermore, women often face inequalities and discrimination in access to health, education, and employment that have negative impact in their lives [1, 2]. In addition, media often portrays women in the stereotyped role whose only responsibility is to look after the family and household chores [2]. Likewise, women have less access and control over financial and physical assets [13]. Similarly, in most of the low economic and tribal families’ women face verbal and physical abuse [8].

#### Gender inequality in health

Gender disparity in health is obvious in Pakistan. Women suffer from neglect of health and nutrition. They don’t have reproductive health rights, appropriate prenatal and postnatal care, and decision-making power for birth spacing those results in maternal mortality and morbidity [13]. Women can not take decision for her and her children’s health; she doesn’t have access to quality education and health services [13, 15]. Furthermore, many papers report son preference [1, 3]. Gender-based violence is also very common in Pakistan that leads to harmful consequences on the health and wellbeing of women [9].

#### Gender inequality in education

Low investment in girls’ education has been reported in almost all the papers reviewed. The major reason for low investment is low returns from girls, as boys are perceived to be potential head of the house and future bread winner [6, 10–13, 15]. One of the case study reports, people believe, Muslim women should be brought up in a way that they can fulfill the role of a good daughter, wife, and a mother; and education can have a “bad influence” to develop these characteristics in women [12]. If girls are educated, they become less obedient and evil and don’t take interest in household chores that is the primary responsibility of her [12]. Moreover, religious leaders have strong authority in rural areas. They often misuse Islamic teaching and educate parents that through education, women become independent and cannot become a good mother, daughter, and a wife. These teachings mostly hinder girl’s education. Other barriers in girls’ education are access to the facility and women’s safety. Five studies reported that most of the schools are on long distances and have co-education system that is perceived as un-Islamic. Parents are reluctant to send their daughters for education as they feel unsafe and threatened [1, 4, 12, 13, 15]. Poverty is another root cause of gender disparity in education, as parents cannot afford the education of their children and when there is a choice, preference is given to boys due to their perceived productive role in future. As a result, more dropouts and lower attainment of education by girls particularly living in rural areas [6–9, 11, 13].

#### Gender inequality in employment

Economic disparity due to gender inequality is an alarming issue in Pakistan. The low status of women in society, home care responsibilities, gender stereotyping, and social-cultural humiliated practices against women are the main hurdles in women’s growth and employment opportunities. Low education of females, restriction on mobility, lack of required skillsets, sex-segregated occupational choices are also big obstacles in the attainment of economic opportunities. Most of the women are out of employment, however those who are in economic stream are facing several challenges [7]. They face discrimination in all layers of the economy. Men are mostly on the leadership positions, fewer females are involved in decision making, wages are low for females if compared with males, workplace harassment and unfavourable work environment is common that hinders long stay in job [1, 7, 8]. Moreover, a study reported that in a patriarchal society very limited number of females are in business field and entrepreneurship. The main hurdles are capital unavailability, lack of role models, gender discrimination in business, cultural and local customs, and lack of training and education [8].

#### Gender biased social norms and cultural practices

The gender discrimination is deeply rooted in the Pakistani society. The gender disparity in Pakistan is evident at household level. It includes Distribution of food, education, health care, early and forced marriages, denial of inheritance right, mobility restriction, abuse, and violence [1, 2, 4, 6, 7, 11]. Furthermore, birth of a boy child is celebrated, and the girl is seen as a burden. Likewise, household chores are duty of a female, and she cannot demand or expect any reward for it. On the other hand, male work has socio-economic value [2, 7, 15]. Furthermore, the female has limited decision making power and most of the decisions are done by male figures in a family or a leader of the tribe or community who is always a male. This patriarchal system is sustained and practiced under the name of Islamic teaching [2, 12, 13]. The prevalence of gender-based violence is also high, in form of verbal abuse, physical abuse, sexual assault, rape and forced sex, etc., In addition, it is usually considered a private matter and legal actions are not taken against it [8] . Moreover, Karo Kari or honor killing of a female is observed in Pakistan. It is justified as killing in the name of honor*.* Similarly, women face other forms of gender-based violence that include: (i) bride price (The family of the groom pay their future in-laws at the start of their marriage), (ii) Watta Satta (simultaneous marriage of a brother-sister pair from two households.), (iii) Vani (girls, often minors, are given in marriage or servitude to an aggrieved family as compensation to end disputes, often murder) and (iv) marriage with Quran (the male members of the families marry off their girl child to Holy Quran in order to take control of the property that legally belongs to the girl and would get transferred to her after marriage) [1, 4, 9, 14, 15]. Furthermore, the women are restricted to choose political career [13].

#### Micro and macro level recommendations

The women should have equal status and participation in all aspects of life that include, health, nutrition, education, employment, and politics [1, 4, 7, 9, 11]. Women empowerment should be reinforced at policy level [1, 7]. For this, constitution of Pakistan should give equal rights to all citizens. Women should be educated about their rights [1, 2, 4, 6, 13–15]. To improve status of women, utmost intervention is an investment in girls education. If women is not educated she cannot fight for her rights. Gender parity can only be achieved if women is educated and allowed to participate in decision-making process of law and policies [4–6, 9, 11, 14]. Similarly, access to health care services is women’s right. Quality education, adequate nutrition, antenatal and post-natal care services, skilled birth attendants, and access and awareness about contraceptives is important to improve women’s health and reduce maternal mortality.

Similarly, women should be given equal opportunities to take part in national development and economic activities of the country to reduce poverty. This is possible through fair employment opportunities, support in women’s own business, equitable policies at workplace and uniform wages and salaries. Besides these, female employees must be informed about their rights and privileges at workplace and employment [1, 7, 8, 11]. Policy actions should be taken to increase the level of women’s participation in economic growth and entrepreneurship opportunities. There should be active actions to identify bottlenecks of gender parity and unlock growth potential of social institutions [5]. Another barrier for women empowerment is threatened and unsafe environment to thrive. There should be policies and legislation to protect women from harm, violence, and honor killing that ensure their health, safety, and wellbeing [4, 12]. Educational institutions and mass media are two powerful sources that can bring change in society. Government must initiate mass media awareness campaign on gender discrimination at household level, educational institutes, and employment sectors to break discriminatory norms of patriarchal society and to reduce the monopoly of males in marketplace. Parent’s education on gender-equitable practices is also important to bring change at the microlevel. It includes gender-equitable child-rearing practices at home including boys mentoring because they think discrimination against females is a very normal practice and part of a culture [3]. There is insufficient data on women’s participation and gender parity in health, education, and employment. Thus, there is a strong need to identify effective interventions and relevant stakeholders to reduce the gender discrimination in Pakistan [5] .

### Findings from primary data collection

The following are the major themes emerged from the primary data collection (Refer Table 3).

**Table 3:** Major themes emerged from the primary data collection

Themes	Categories
Theme: 1 Perception of women regarding gender discrimination in society	a) Woman as a sexual object
	b) Women as dependent beings
	c) Women’s autonomy
	d) Males as an identity for females
	e) Child’s upbringing responsibility
	f) Unrecognized contribution of women
	g) Gender differences in daily activities
	h) Deprivation of women’s rights
Theme 2: Perception of men regarding gender discrimination in society	a) Male Dominance
	b) Preference of male child
	c) Lack of communication among husband and wife
	d) Men are protectors
Theme 3: Factors influencing gender discrimination.	a) Role of family head
	b) Media influence

#### Theme 1: perception of women regarding gender discrimination in society

##### Woman as a sexual object

Female participants highlighted that they are seen as “sexual objects” and “a mean of physical attraction” which prevents them from comfortably leaving their homes. One female participant explained this further as,

*“We are asked to stay inside the house because men and boys would look at our body and may have bad intentions about us”* (Adolescent girl, FGD).

Male participants echoed this narrative as they agreed that women are judged by their physical appearance, such as the shape of their bodies. A male participant stated,

“*Woman is a symbol of beauty and she's seen by the society as the symbol of sex for a man"* (Male HCP, IDI).

A male participant reported,

*“Women should cover themselves and stay inside the house”* (married man, FGD).

One female participant verbalized,

“*We have breasts, and therefore, we are asked to dress properly".*(adolescent girls, FGD).

Another stated,

“*Girls are supposed to dress properly and avoid eye contact with boys while walking on the road”* (adolescent girls, FGD).

##### Women as dependent beings

One of the major study findings suggests the idea that women must be “helped” at all times, as they are naturally dependent upon other persons to protect them. One participant stated,

*“If a woman is alone, she is afraid of the man's actions*” (adolescent girl, FGD).

Some female participants, however, agreed with this statement to some extent because they felt that men help women to fit into society. Oftentimes, judgment is passed for women without an accompanying male. Participants verbalized that wife cannot survive without husband and similarly daughter cannot live without her father. One participant mentioned,

*“We are only allowed to go out when we have our father or brothers to accompany us”* (Adolescent girl, FGD).

Other participants agreed with the sentiment differently. Since it is implied that men easily get attracted to women, having a male figure with female will protect her from naturally prying eyes. However, if she cannot be accompanied by a male, she must protect herself by covering fully and maintain distance with males.

##### Women’s autonomy

Female participants, especially young adolescent girls, shared how restrictions have affected their livelihoods. Participants expressed how easy it is for males to gain permission and leave the house, while females often have series of obstacles in front of them. A young girl stated,

“*There are lot of constraints when we see women in our culture. They must take care of everything at home, yet they must get everybody's permission to go five minutes away. Whereas a boy can go out of town and that too, without anyone’s permission. Looking at this, I wish I were a boy. I'd go wherever I want, and I could do whatever I want”* (adolescent girl, FGD).

##### Males as an identity for females

Women are often identified through a prominent male figure in their life and are not considered to have individual personalities and identities. A female participant mentioned that,

*“Woman is someone having a low status in society. People know her through their husband or father name”* (married women, FGD).

##### Child’s upbringing responsibility

Culturally, it is expected from the female members of the family, often mothers, to rear children and take care of their upbringing. Male members, mainly fathers, are expected to look after finances. Thus, mothers usually take a greater portion of responsibility for child’s upbringing and blame in case of misconduct. A married woman explained that,

*"If a girl does something, the mother is blamed for that. Even in our house, my mother-in-law talks to my mother if I argue or refuse for anything. This is the culture in my maiden home as well"* (Married Woman, FGD).

##### Unrecognized contribution of women

Many female participants verbalized their concern for disregard they receive from their families despite contributing significantly. Women who perform major roles in maintaining the family and household chores are not recognized for their efforts. By doing cleaning, cooking and other duties, they keep family healthy and help keep costs low. One participant mentioned,

*“If women don’t clean the house, it is extremely dirty. If women do not rear children, no one else would do it. We do so much for the family”* (married woman, FGD).

##### Gender differences in daily activities

Both men and women struggle with self-expression as certain expectations from both genders hold people back from expressing their views and opinions. Men, for example, as indicated by participants, are expected to remain firm in challenging situations and not show emotions. Even for hobbies, participants shared that, parks and recreational activities are geared towards young boys and men, while girls and women are given more quiet and indoor activities. A female participant verbalized that,

“*Boys have a separate area where they play cricket and football daily but for girls like us, only indoor activities are arranged”* (adolescent girl, FGD).

In places where males and females freely mix or live closely in one area, people often find themselves taking extra precautions in their actions, as to not be seen disgraceful by the community. One female participant reported,

“*Two communities are residing in our area. Events for females, such as sports day, are very rarely arranged. Even then we cannot fully enjoy because if we'll shout to cheer up other players, we would be scolded as our community is very cautious for portraying a soft image of females of our community*” (adolescent girl, FGD).

Another participant stated that,

“*After prayers, we cannot spend time with friends as people would point that girl and say that she always stays late after prayers to gossip when she is supposed to go home*” (adolescent girl, FGD).

##### Deprivation of women’s rights

A woman’s liberty has always struggled to be accepted and males are always favoured. Thus, women are given lower status. Participants highlighted that, in general, men are seen as superior to women. One participant stated,

“*Men are the masters of women…”* (FGD married women).

On the other side, male suppress female liberty and women are unaware of their rights leaving them vulnerable to deprivation. A female participant explained,

*“Women do not dominate society that's why people take away their rights from them”* (married woman, FGD).

Female participants also shared that they see men as strong and dominant personalities, making them better decision makers regarding health care acquisition, family income, availing opportunities and producing offspring. One female participant verbalized,

*“If there's one egg on the table and two children to be fed, it is considered that males should get it as it is believed that males need more nutrition than us”* (HCP, IDI).

Another reported that,

*“There is a lack of equal accessibility of health care facilities and lack of employment equality for women”* (HCP, IDI).

#### Theme 2: perception of men regarding gender discrimination in society

##### Male dominance

Inferiority and superiority are common phenomenon in Pakistan’s largely patriarchal society. This allows men to be seen as dominant, decision-maker of family and the sole bread winner. Women, however, are caught in a culture of subordination to men with little power over family and individual affairs. A female participant said,

*“If we look at our society, men are dominant. They can do anything while a woman cannot, as she is afraid of the man's reactions [gussa] and aggression”* (adolescent girl, FGD).

While another reported,

*"In our society, husband makes his wife feel his superiority over her and would make her realize that it is him, who has all the authority and power”* (married woman, FGD).

##### Preference for male child

There is often an extreme desire for birth of sons over daughters, which adds to the culture of gender discrimination in Pakistan. Male children are important to the family as they often serve their parents financially, once they are able. This is one of the main reasons that parents are more inclined towards birth of a male child rather than female. Consequently, education is prioritized for male children. Female participants expressed that their desire for a male child is to appease their husband’s family and reduce the pressure on her to fit in the house. According to a female participant,

*“When my son was born, I was satisfied as now nobody would pressurize me. I noticed a huge difference in the behavior of my in-laws after I gave birth to my son. I felt I have an existence in their family”* (married woman, FGD).

Participants highlighted that, women who have brothers are often more protected. According to a young participant,

*“Brothers give us the confidence to move within the society because people think before saying anything about us”* (adolescent girl, FGD).

##### Lack of communication among husband and wife

Married couples often lack communication and rarely discuss important matters with each other. Men often choose not to share issues with their wives as they believe they are not rational enough to understand the situation. A male participant stated,

“*Women are so sensitive to share anything. They can only reproduce and cook food inside the home”* (married man, FGD).

##### Men are protectors

Many female participants considered men as a source of protection, as they manage finances and ensure safety of family members. They feel confident in man’s ability to contribute to their livelihoods. One participant mentioned,

*“We go out when we have our father or brothers to accompany us”* (Adolescent girl, FGD).

Another highlighted,

*“Men are our protectors. We can only survive in the society because of them”* (Married woman, FGD).

#### Theme 3: factors influencing gender discrimination

##### The role of family head

A tight-knit family situation, difference of opinions, cultural values and generation gap can highly affect one’s view on gender. Participants highlighted the role of elders in the family who often favor their sons and male family members. Married women expressed that daughter in-laws often struggle to raise their voice or express their concerns in such family situation. One participant mentioned,

*“We don’t take decisions on when to have the child or what method needs to be used for family planning. Our mothers-in-law decide and we must obey”* (married woman, FGD).

The family system that often includes three generations living closely, allows traditional norms to carry forward, as opposed to a typical nuclear family. This includes attire, conduct, and relationships. One participant mentioned,

*“I live with my mother-in-law. I must cover my head whenever I had to leave the house”.* (Married woman, FGD).

##### Media influence

Media plays an important role in disseminating gender awareness. For example, advertisements of cooking oils and spices usually show young girls helping their mothers in kitchen, while men and boys are observed enjoying something else or not present. These short advertisements are impactful in perpetuating gender conduct solely for societal acceptance. One participant verbalized,

*“Every household has a radio, on which different advertisements are going on. People get messages through media”* (married man, FGD).

## Discussion

The study reveals that women are seen as sexual objects and therefore confined to their homes. Women are often judged on their physical appearance that hinders their autonomy in various aspects of life. Many women face difficulties in leaving their homes alone and require protection from men [3]. Men are, therefore, labeled as protectors while women are regarded as dependent beings who need man’s identity. The role of men inside the house is identified as authoritative, while women need approval from male because they are considered incapable of making appropriate decisions. Women are caretaker of their families and have primary responsibility of husband, children, and in-laws. However, these contributions are mostly unnoticed. These gender power differentials are so strong in households, that many women do not know their rights. Women comply with societal and cultural values that force them to become lesser beings in the society. Girls in society grow up and eventually adopt the traditional role of women [8]. Increased education and awareness level among communities can improve status of women in the Pakistani society [3].

Moreover, males have dominant role in the society [1]. Likewise, there is discrepancy in power structures between male and female in the family system that often leads to lack of communication especially between married couples as husbands do not share concerns with their wives nor ask for their advice, considering women incapable to understand anything [5].

Furthermore, a common phenomenon observed in the Pakistani society, is the strong desire for a male child, while the birth of a female child is mourned [5]. Girls are seen as a liability, while the birth of a male child is celebrated as it is believed that males will be the breadwinner of the family in the future [5]. Thus, preference for a male child leads to illegal termination of pregnancies with female fetuses in many situations [9]. In addition, some of the studies suggest that the preference for a son is significantly high in low socioeconomic areas if compared with the middle and upper ones. Men are seen as economic and social security providers of the household. Therefore, men are tagged as manhood in the society as it is considered that hierarchal familial structures are produced from them, and all powers are attributed to men. This increases the disparity of roles between men and women leading to gender discrimination [5]. Our study also reveals that media has important influence towards gender discrimination. It is commonly observed in the Pakistani TV advertisements, that household chores are mostly performed by women while men have professional roles in the society [6].

Thus, lack of female autonomy and empowerment are recognized as the major reasons of discrimination of women in our society. They do not have the means to participate in society, neither they are allowed to speak against traditions. Therefore, interventions are required to increase female autonomy and decision-making capacity. The other significant contributor to gender discrimination is male dominance, which must be brought down to empower women. To reduce this, communication is key between spouses, family members and community members. Gender discrimination has greater influence at different levels of Pakistani society. Certain schools and television advertisements portrays stereotypes, such as allowing boys to be active outdoors and forcing girls to remain indoors. Therefore, media channels and other public systems such as healthcare facilities and schooling systems must promote gender equity and equality. In terms of Sexual and Reproductive health (SRH), the health care facilities should play an important role in providing knowledge and effective treatment to both males and females. The SRH related services are often compromised for people due to lack of resources, staff, and attention. Schools and communities should play an important role in creating SRH related awareness among youth and adults that include puberty, pregnancy, and motherhood. SRH should also be made part of curriculum in educational institutes.

The use of group interviews allowed rapport development with communities. With multiple people present sharing similar views, many were inclined to give purposeful answers and recommendations regarding gender roles in communities. Based on previous literature searches, this study, to the best of our knowledge, has not been published in Pakistan at the community level. No other study explores the views of Pakistanis on gender discrimination with inclusion of multiple community groups and across multiple districts. In limitations, due to the topic’s sensitive topic, may have held back participants from answering fully and truthfully. Thus, considerable time was taken to develop trust and rapport. Therefore, it is possible that some study subjects might not have answered to the best of their ability. Furthermore, challenges were faced due to the COVID-19 pandemic and extreme weather conditions in some areas, as some participants could not reach the venue. Also, the lockdowns following the pandemic made it very difficult to gather 10–12 people at one place for the FGDs. Interviews could not be done virtually as the information was very sensitive.

## Conclusion

Gender roles in Pakistani society are extremely complex and are transferred from generation to generation with minimal changes since ages. This study reveals some of the factors due to which women in Pakistan face gender discrimination. The cultural and societal values place women in a nurturing role in the Pakistani society. Through reinforcement of these roles by different family members, as well as by the dominant men in the society, women face adverse challenges to seek empowerment that will help them defy such repressive roles assigned to them. Gender discrimination is evident in public institutions such as healthcare facilities and schooling systems. Thus, administrative reorganization and improved awareness in the healthcare facilities, and appropriate education in schools for boys and girls will help decrease gender discrimination in the Pakistani societies.

## Data Availability

On request, the data will be available by hiding the IDs. Though we have already provided the transcripts, yet there is a need of further information then kindly contact the corresponding author. Dr. Tazeen Saeed Ali: tazeen.ali@aku.edu.

## Abbreviations

AKF: Aga Khan Foundation
PRISMA: Preferred Reporting Items for Systematic Reviews and Meta-Analyses
STROBE: Reporting of Observational studies in Epidemiology
IPV: Intimate Partner Violence
HCP: Healthcare Professionals
LHV: Lady Health Visitors
LHWs: Lady Health Workers
CMWs: Community Midwives
IDIs: In-Depth Interviews
FGDs: Focus Group Discussions
HODs: Heads of the Departments
SRH: Sexual and Reproductive health
UNFPA: United Nations Population Fund
ERC: Ethics Review Committee
